# Meeting the health needs of forcibly displaced populations: translating evidence into implementation via the WHO global research agenda on migration and health

**DOI:** 10.1093/eurpub/ckac129.114

**Published:** 2022-10-25

**Authors:** M Orcutt

**Affiliations:** Health and Migration Programme, WHO, Geneva, Switzerland

## Abstract

In this presentation, Dr Orcutt, will outline the health system responses needed to respond effectively to the health needs of forcibly displaced populations - from initial health assessments to the longer term - and the role that research can play in ensuring evidence-informed policies and practice. She will present the main areas of the WHO's new global research agenda on migration and health and outline how translating evidence into implementation is essential to improve the health of migrants.

